# Truncated Cotton Subtilase Promoter Directs Guard Cell-Specific Expression of Foreign Genes in Tobacco and Arabidopsis

**DOI:** 10.1371/journal.pone.0059802

**Published:** 2013-03-29

**Authors:** Lei Han, Ya-Nan Han, Xing-Guo Xiao

**Affiliations:** State Key Laboratory of Plant Physiology and Biochemistry, College of Biological Sciences, China Agricultural University, Beijing, China; New Mexico State University, United States of America

## Abstract

A 993-bp regulatory region upstream of the translation start codon of subtilisin-like serine protease gene was isolated from *Gossypium barbadense*. This (T/A)AAAG-rich region, *GbSLSP*, and its 5′- and 3′-truncated versions were transferred into tobacco and Arabidopsis after fusing with *GUS* or *GFP*. Histochemical and quantitative GUS analysis and confocal GFP fluorescence scanning in the transgenic plants showed that the GbSLSP-driven *GUS* and *GFP* expressed preferentially in guard cells, whereas driven by *GbSLSPF2* to *GbSLSPF4,* the 5′-truncated *GbSLSP* versions with progressively reduced Dof1 elements, both *GUS* and *GFP* expressed exclusively in guard cells, and the expression strength declined with (T/A)AAAG copy decrement. Deletion of 5′-untranslated region from *GbSLSP* markedly weakened the activity of GUS and GFP, while deletion from the strongest guard cell-specific promoter, *GbSLSPF2*, not only significantly decreased the expression strength, but also completely abolished the guard cell specificity. These results suggested both guard cell specificity and expression strength of the promoters be coordinately controlled by 5′-untranslated region and a cluster of at least 3 (T/A)AAAG elements within a region of about 100 bp relative to transcription start site. Our guard cell-specific promoters will enrich tools to manipulate gene expression in guard cells for scientific research and crop improvement.

## Introduction

Stomata are the specialized structure of plant epidermal cells, containing a pair of guard cells and a pore between them. They control gas exchange between plant and atmosphere, taking an important part in photosynthesis, respiration and transpiration. Their density and pore size greatly affect the gas exchange rate and loss of water. Under normal environmental conditions, a given plant can make balance between the CO_2_ uptake for photosynthesis and the loss of water for transpiration by changing stomatal pore size [1]–[3]. In other hand, the stomatal pore size is also regulated by both biotic stimuli and abiotic stimuli such as leaf excision [4], pathogens [5], [6], light [7], [8], CO_2_ [5], [1], [9], ozone (O_3_) [10], temperature [11], H_2_S [12], humidity [13], abscisic acid (ABA) and other plant hormones [14]–[16], and combination of abiotic factors In some case, the biotic and abiotic stimuli cross-regulated stomatal pore size [17]. However, no mater the stomatal pore size change is actively regulated by plant itself or passively regulated by biotic and/or abiotic stimuli, it is the guard cells that carry out these regular “orders” and make stomata movement appropriately. Consequently, the guard cells are key regulatory elements in the control of photosynthesis and transpiration [18]. Therefore, these “orders-receptor(s)” and “orders-executor(s)” must be guard cells-specific.

Search of these receptor(s) and/or executor, besides proteins involved in the growth and development of guard cells themselves, led to identification, isolation and functional analysis of a grand body of guard cells-specific and/or preferred genes and promoters [19], [20]. *Akt1* [21], *Kat1* [22], rha1 [23], dehydrins [24], *CHX20* [25], *MYB60* [26], *SLAC1* [27], [28], *ALMT12* [29], *TPC1* [30] and *ROP11* [31] from Arabidopsis and *Kst1* [32] from potato are of the representative genes. As for the promoter, *Kat1* [33], *Kst1* [34], [35]) *Abh1* [36], *Chl1* [37], *Rac1* [38], *Osm1* [39], *Ost1* [40], *MYB60* [26], [41], [42], *AtCHX20* [25], [43], *SLAC1* [27], [28], *PDR3* [26] and *ROP11* [31] are in the list. Unfortunately, a grand majority of promoters elucidated are not strictly guard cell-specific, but guard cell-preferred, strongly or modestly. Recently, three promoters from Arabidopsis, *pGC1* [44], *CYP86A2* [45] and *MYB60* [42], [46], were reported to drive guard cell-exclusive expression of genes in transgenic plants. Interestingly, although most native promoters from guard cell-preferred genes were not strictly guard cell-specific, some truncated promoters from these genes and even from genes not guard cells-preferred were strictly guard cell-specific. Muller-Rober et al [32] demonstrated that a fragment of ca. 300 bp left by 5′-deleting an ADP-glucose pyrophosphorylase promoter from potato could drive GUS reporter gene to express exclusively in the guard cells of transgenic potato and tobacco plants. This truncated promoter was used to drive strictly guard cell-specific expression of *AtALMT12* in Arabidopsis successfully [29]. Guard cell-specific gene expression was found to be controlled principally by Dof1 protein-targeted *cis*-acting element, 5′-(T/A)AAAG-3′, in particular TAAAG, proximal to TATA-box in potato *KST1* promoter [35]. This element, (T/A)AAAG, was later successfully used to construct and express a “tailor-made” drought-inducible guard cell-specific promoter *DGP1* [47]. In this inspiration, we scanned DNA databases available with (T/A)AAAG as probe to identify and then clone guard cell promoters for further use in molecular engineering of guard cells and hence increasing the adaptation of crop plants to environment stress.

Our scanning with the probe (T/A)AAAG found large numbers of promoter candidates (Unpublished). Among them, the promoters of subtilisin-like serine protease (subtilase) genes attracted us most, because of some them involved in epidermal surface formation such as *AtALE1* [48] and guard cell development including stomatal density and distribution such as *AtSDD1* [49]–[51]. We targeted the promoters of cotton subtilisin-like serine protease genes and cloned a 5′-flanking fragment of 993 bp upstream of the translation start codon “ATG” from sea island cotton (*Gossypium barbadense*) [52]. Here we show that this (T/A)AAAG-rich fragment, *GbSLSP*, directed high level of guard cell-preferred expression of both GUS and GFP reporter genes in transgenic tobacco and Arabidopsis. We demonstrate that several 5′-end truncated versions of *GbSLSP* could drive the reporter genes to express exclusively and strongly in the guard cells. Finally, we reveal that the guard cell specificity of 5′-truncated *GbSLSP* is coordinately controlled by 5′-untranslated region (5′-UTR) and a cluster of at least 3 *cis*-acting elements (T/A)AAAG within a region of about 100 bp relative to transcription start site. Our results will provide an additional tool in getting strictly guard cell-specific promoters and thus in the improvement of crops adaptation to environment via gene engineering of guard cells.

## Materials and Methods

### Plant Material and Growth Conditions

Seeds of sea island cotton (*Gossypium barbadense* L. cv. SHZ2-214) were kindly provided by Dr. J.B. Zhu of University of Shehezi, China. Cotton and tobacco (*Nicotiana tabacum* cv. NC89) plants were grown in a greenhouse at 25±2°C, and *Arabidopsis thaliana* ecotype “Columbia” at 22±1°C under 16-h light/8-h dark cycle in a culture room.

### Promoter Isolation and Plant Expression Vector Construction

Sea island cotton genomic DNA was extracted from fresh young leaves with improved CTAB method [53]. The 5′-flanking region of about 1000 bp upstream of the translation start codon “ATG” of a cotton subtilisin-like serine protease gene [54], [55] was isolated by using polymerase chain reaction (PCR) with primer pair 5′-AAGCTTACAACTTTTCTCTACCAATCA-3′/5′-GGATCCGCTAGAGAAAAATGGGAAGGTGAG-3′ (*Hin* dIII and *Bam* HI restriction sites added were underlined respectively). The PCR products were ligated in pBS-T vector (Qiagen, China), and then sequenced to check the identity after size verification by *Hin* dIII-*Bam* HI digestion. The expected fragment, named “*GbSLSP”* or simply “F1”, was designed as full length promoter in this study. From this full-length promoter, sets of progressive 5′-deletion and 3′-deletion fragments were generated by PCR using specific primers (Table 1). All fragments obtained were cloned into pBS-T vector and sequenced as described above.

**Table 1:** Oligonucleotide primers used for PCR cloning and deletion of GbSLSP promoter.

Primer name	Primer sequence (5′ to 3′)*
Forward	
SLSPFW1	AAGCTT ACAACTTTTCTCTACCAATCA
SLSPFW2	CAATATGAAA***AAGCTT***GAGTGC
SLSPFW3	AAGCTT ATTTTGGAAGATGAC
SLSPFW4	AAGCTT CTTTACATGCATCATGTGATCG
SLSPFW5	AAGCTT ATCGTGGGGGACCCGAAACTTGGCATAC
Reverse	
SLSPRW1	GGATCC GCTAGAGAAAAATGGGAAGGTGAG
SLSPRW2	GGATCC GTGG TTGGATGAGACT

* Underlined are *Hin* dIII and *Bam* HI recognizing sites added at the forward and reverse primers, respectively. The bold italic is the native *Hin* dIII recognizing site.

The sequencing-verified “promoter” fragments were isolated from their correspondent pBS-T with *Hin* dIII-*Bam* HI and individually cloned into a binary vector pBI121 (Clontech) to replace CaMV 35S (35S) promoter, which gave rise to pGbSLSPn-GUS vectors (here n = F1 to F2-sh). To construct pGbSLSPn-GFP vectors, the GUS coding sequence in the pGbSLSPn-GUS was replaced by GFP coding sequence PCR-amplified from pCAMBIA1300. During PCR amplification, *Bam* HI and *Sac* I restriction sites added to the 5′ and 3′ ends of the GFP.

### Plant Transformation and Growth Conditions

All constructs described in the previous section were transferred to *Agrobacterium tumefaciens* strains GV3101 and LBA4404 for transformation of Arabidopsis and tobacco, respectively. *Agrobacterium*-mediated transformation of Arabidopsis (ecotype “Columbia”) and tobacco (*N. tabacum* cv. “NC89”) was conducted by using methods of floral-dip [56] and leaf disc co-culture [57], respectively.

Tobacco transformants were selection on MS medium containing 50 mg/L of kanamycine (Kan) and 500 mg/L of cefotaxime under 16-h light/8-h dark cycle at 24°C ±1°C in a culture room. The Kan-resistant shoots were rooted in MS containing 100 mg/L of Kan, and resulting plantlets were then transplanted in pots in a greenhouse. For selection of Arabidopsis transformants, the seeds of floral dip-transformed plants were surface-sterilized in dilute bleach (0.5% NaClO) for 10 min and then with 75% ethanol for 30 s, rinsed five times with sterile distilled water. The sterilized seeds were then germinated on MS medium containing 50 mg/L of Kan, stratified for 2 d at 4°C and then placed under 16-h light/8-h dark cycle at 22°C ±1°C in a culture room. The Kan-resistant seedlings were transplanted in pot and grew in the culture room.

### Analysis of GUS and GFP Expression

Histochemical staining and quantitative analysis for GUS activity in the transgenic plants were performed as described by Jefferson et al. [58]. Briefly, for GUS staining, samples were incubated in GUS staining solution (50 mM phosphate buffer, pH6.7, 1 mM EDTA pH8.0, 0.2% (V/V) Triton-100, 1 mM K_3_FeCN_6_, 1 mM K_4_FeCN_6_, 0.5 mg/mL 5-bromo-4-chloro-3-indoxyl-D-glucuronic acid (X-gluc)) at 37°C for 12 to 16 h. After staining, the samples were cleared with 70% ethanol for more than 1 h at room temperature, and then photographed by using an Olympus SZX16-DP72 stereomicroscope. For quantitative GUS activity assay, the samples were prepared as previously described [59], and the enzymatic reaction was carried out in a reaction volume of 500 µl and at 37°C. At zero time, an aliquot of 50 µl reaction solution was taken out and added to 450 µl 0.2 M Na_2_CO_3_ and the same manipulation was performed at subsequent times 10, 20, 30, 45, 60 min. The GUS activity was detected in HITACHI F-4500 spectrofluorometer with excitation at 365 nm and emission at 455 nm, and expressed as nmol of 4-methylumbelliferone (MU) produced per mg protein per min.

Detection of GFP fluorescence in the leaves of transgenic plants was carried out using Carl Zeiss LSM510 laser scanning confocal imaging system at 488 nm excitation, and emission band width of 505–530 nm. For chlorophyll detection, the excitation was at 543 nm and emission at LP650 nm.

## Results

### *GbSLSP* has Multiple Copies of Guard Cell-specific *cis*-element TAAAG and Alike Elements

**Figure 1:**
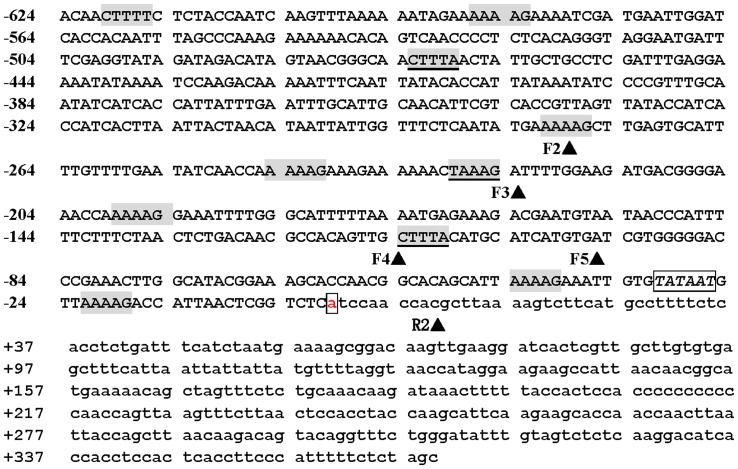
Nucleotide sequence of the 5′-flanking region of GbSLSP gene. Nucleotides are numbered on the left with the transcription start site designated as +1 which is white-boxed. The 5′-UTR is in lower case letters. The TATA-box is in italic letters and white-boxed. The DOF1-binding sites AAAAG are grey-boxed, and TAAAG, grey-boxed and underlined. The deletion positions are indicated with arrowheads behind the short name of forward (F2 to F5) and reverse (R2) primers.

A 993-bp promoter region upstream of the translation start codon “ATG” of a subtilisin-like serine protease gene from sea island cotton was PCR-amplified by using primer pair SLSPFW1/SLSPRW1 (Table 1), and this region consisted of a regulatory fragment of 624 bp and a 5′-UTR of 369 bp based on online promoter prediction and comparison with reported GhSCFP (54, 55). Online analysis using SoftBerry (http://linux1.softberry.com) and PLACE (http://www.dna.affrc.go.jp/htdocs/PLACE/) [60] of the regulatory fragment revealed the presence of 1 TATA box (−31) and 10 Dof protein-targeted *cis*-acting elements “(T/A)AAAG” (Fig. 1). Among the *cis*-elements, three were guard cell-specific ones (TAAAG) as defined by Plesch et al. [35], one in sense strand (−229) and two in antisense one (−114, −483), and the rest were TAAAG-like element, “AAAAG”, 6 in sense strand and 1 in the antisense (Fig. 1). This promoter region was designated as “*GbSLSP*”, simply called “F1” and used as full length promoter for coming experiments.

### *GbSLSP* Directed Strong Guard Cell-preferred Expression of GUS and GFP Reporter Genes in Transgenic Tobacco Plants

In order to investigate the driving pattern and strength of *GbSLSP*, we first constructed GbSLSP::GUS cassette (pGbSLSP-GUS) by cloning the *GbSLSP* into binary vector pBI121 to replace *CaMV 35S* promoter and obtained more than 30 independent transgenic tobacco plants via *Agrobacterium*-mediation transformation. Ten plants with expected strong and sharp PCR-amplified band (Figure not shown) were used for GUS expression analysis.

**Figure 2:**
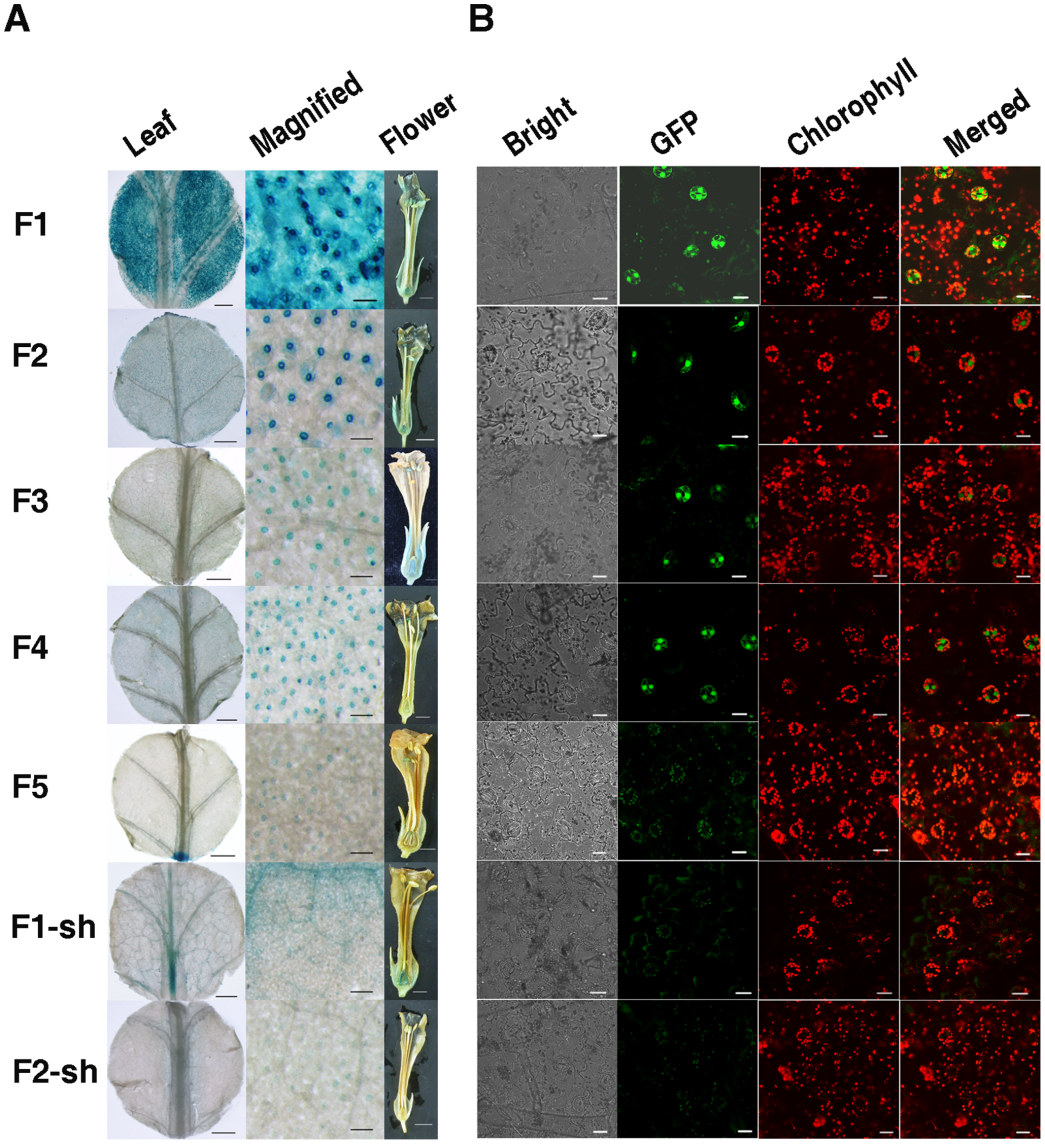
Histochemical GUS staining and Confocal GFP fluorescence scanning of transgenic tobacco T0 plants transformed with the full length promoter GbSLSP and its 5′- and 3′-truncated versions. A, Histochemical GUS staining of young leaves and flowers. B, Confocal GFP fluorescence scanning of young leaves. F1: Transformed with full-length GbSLSP(F)::GUS/GFP. F2–F5: Transformed with GbSLSPFn:: GUS/GFP, where n = 2 to 5. F-sh: Transformed with full-length GbSLSP(F)-sh::GUS/GFP. F2-sh: Transformed with full-length GbSLSPF2-sh::GUS/GFP.

**Figure 3:**
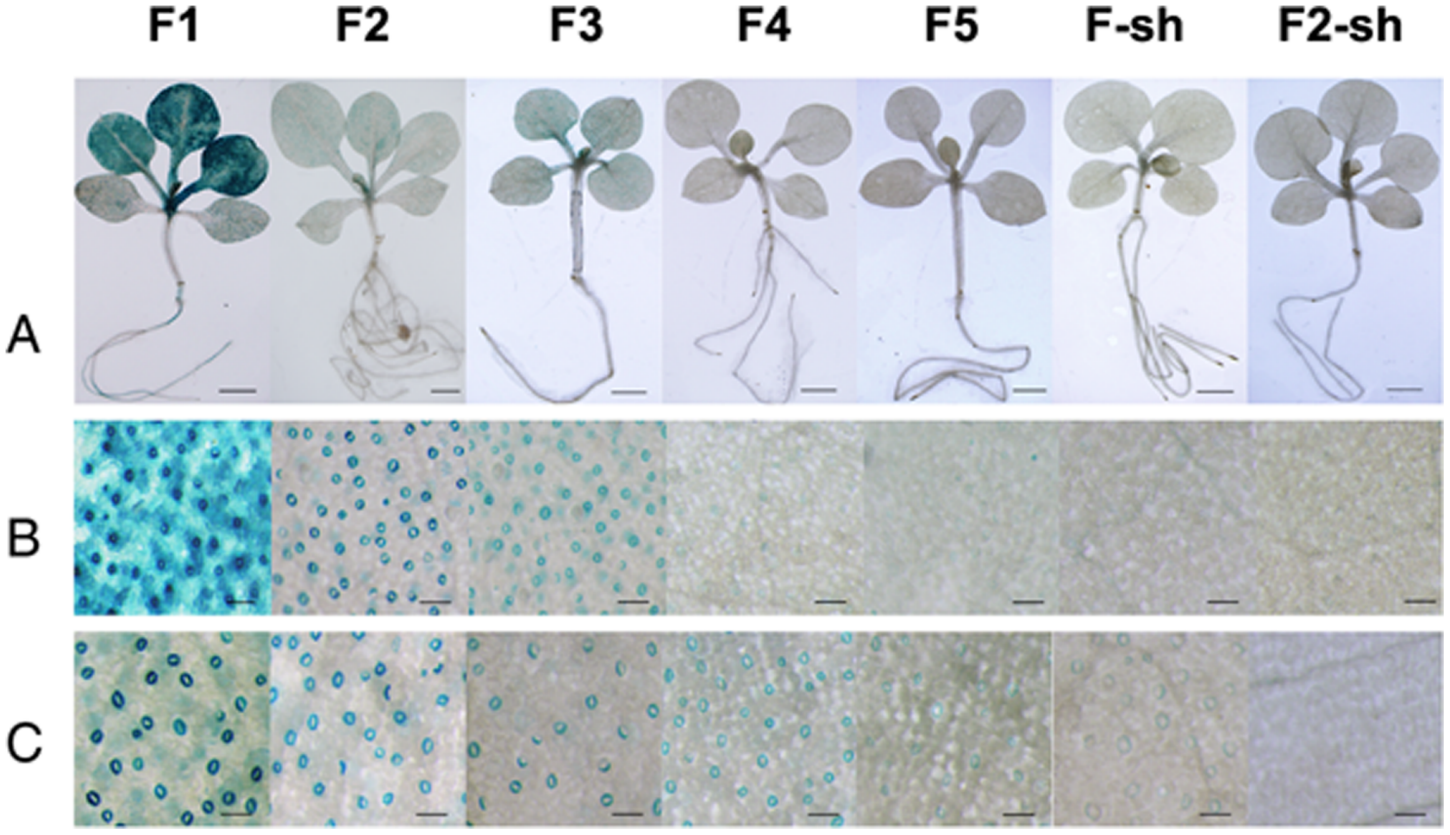
Histochemical GUS staining of T1 seedling of tobacco transformed with the full length promoter GbSLSP and its 5′- and 3′-truncated versions. A, Whole seedlings, scale bars = 0.2 mm. B, Rosette leaf zones, scale bars = 0.1 mm. C, Cotyledon zones, scale bars = 0.1 mm.

Histochemical GUS staining of T0 GbSLSP::GUS-transgenic tobacco showed that GUS gene was expressed very strongly in guard cells, strongly in mesophyll cells adjacent to the guard cells, less-strongly in veins and trichomes of the leaves, moderately in ovary wall and slightly in sepal, stigma and in some anthers (Fig. 2A: F1). The overall strength of GUS expression driven by *GbSLSP* in the leaf was approximately 70% of that driven by *CaMV 35S* (data not shown). In the Kan-resistant T1 seedlings at 3–4-leaf stage, GUS expression pattern and strength were similar to their parental lines in leaves (Figs. 3A & 3B: F1), but in guard cell-specific manner in cotyledons (Fig. 3C: F1). Very weak GUS staining also appeared in the lower part of the root vascular system (Fig. 3A: F1).

In order to verify whether the expression patterns of GbSLSP observed using GUS as reporter were an accurate representation of the *GbSLSP*, we replaced the *GUS* with *GFP* in the vector pGbSLSP-GUS and generated more than 20 independent GbSLSP::GFP-transgenic tobacco plants also by *Agrobacterium*-mediation transformation. Ten independent T0 GbSLSP::GFP transformants at 5–6-leaf stage were selected for analysis of GFP expression pattern in the young leaves. As showed in Figure 2B:F1, the GFP expression pattern looked like the GUS expression as showed in Fig. 2A:F1 and the transgenic lines displayed strong GFP signals in guard cells and much weaker GFP signals in the cells adjacent to the guard cells in the leaves.

### 5′-truncated Versions of *GbSLSP* Drove GUS and GFP Reporter Genes to Express Specifically in Guard Cells of Transgenic Tobacco Plants

**Figure 4:**
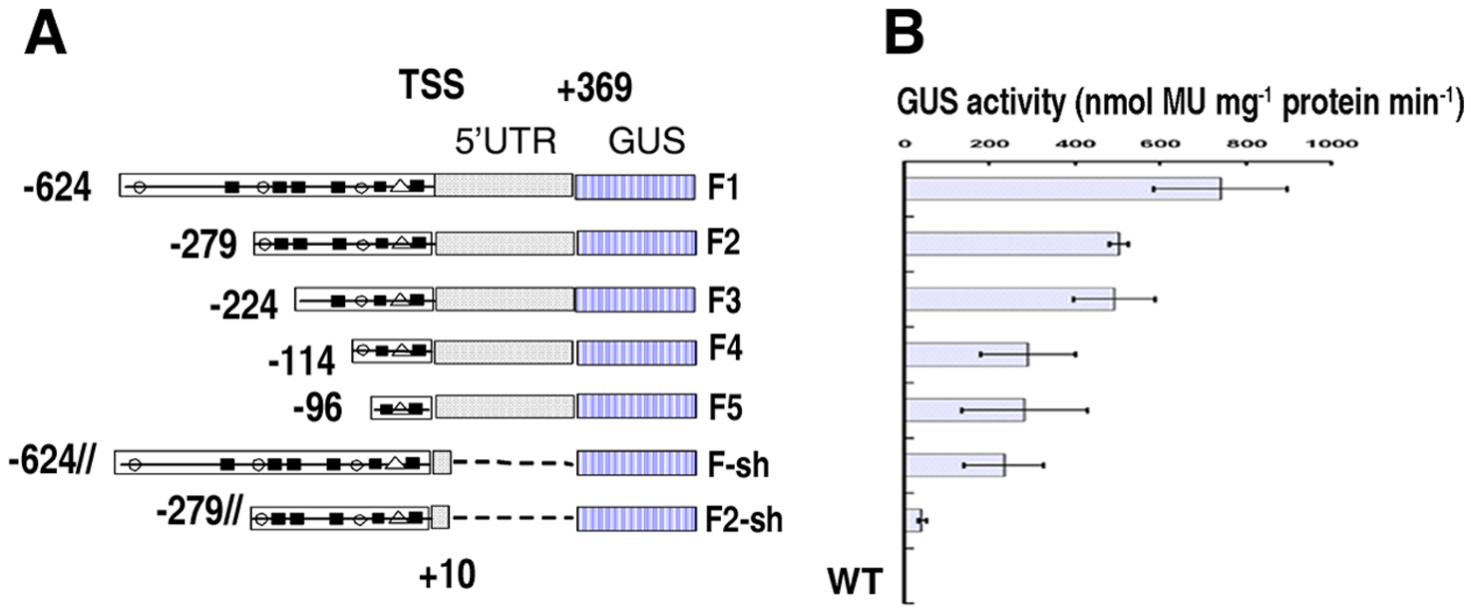
Schematic presentation of the GbSLSP promoter 5′-and 3′-deletion series. A, Schematic diagrams of deletion series constructs. Solid round (•), the (T/A)AAAG elements on sense strand (5′ to 3′); Solid square (▪), (T/A)AAAG elements on antisense strand (3′ to 5′); White triangle (△), TATA-box. B, Quantitative analysis of GUS activity from the deletion series in T0 transgenic tobacco leaves. Values represent mean and standard error.

As analyzed in the previous section, the *GbSLSP* contained several copies of guard cell-specific *cis*-element TAAAG and TAAAG-like element, the targeted sites of Dof1 protein. To gain insight into the functional role of (T/A) AAAG elements in the expression pattern of the promoter, we made a progressive 5′-deletions of *GbSLSP* by PCR using specific primer pairs SLSPF2-SLSPFW5/SLSPRW1 (Table 1) with consideration of progressively reducing the copy number of (T/A)AAAG elements. The set of 5′-deletion generated four 5′-truncated promoters (Fig. 4A: F2 to F5). The *F5* (−96 to +369) contained 2 (T/A)AAAG elements in sense strand, and from *F5* to *F3*, one more (T/A)AAAG element in sense or antisense strand was presented near 5′-end. For *F2*, it contained 3 more (T/A)AAAG elements than *F3*, 2 in sense strand and 1 in antisense strand.

Binary vectors GbSLSPFn-GUS and GbSLSPFn-GFP (n = 2 to 5) (Fig. 4A) were constructed and *GbSLSPFn::GUS-* and *GbSLSPFn::GFP*-transgenic tobacco plants (each construct with more than 30 independent transformants) were generated with the same methods used for the full length promoter *GbSLSP* as described above.

In the leaves of tobacco plants transformed by 5′-truncated GbSLSP promoter *F2*, *F3* or *F4*, the GUS expression pattern was similar and GUS staining was observed exclusively in guard cells, although the staining strength varied with copy number of (T/A)AAAG motif and/or length of the promoter (Fig. 2A: F2 to F4). The *F2* which contained 7 copies of Dof motif had the strongest GUS staining, whereas the *F4* that possessed only 3 copies of Dof motif, had much weaker one. In *F5*-transgenic tobacco leaves, less than half of plants showed very week GUS staining exclusively in some guard cells, and the rest displayed very week and diffused GUS staining in the cells other than guard cells (Fig. 2A: F5). This GUS staining strength was confirmed by GUS quantitative assay (Fig. 4B). In comparing with full length promoter *GbSLSP (F1)*, the 5′-truncated ones gained guard cell-specificity, but lost part of driving power, even *F2* (Fig. 2A: F2–F5 vs. F1; Fig. 4B).

In the flowers of 5′-truncated GbSLSP-transformants, the GUS expression pattern was varied depending on the length or (T/A)AAAG copy number of the promoter. The *F2* retained the expression pattern and strength of the full-length promoter, whereas *F4* and *F5*, only very week GUS staining could be detected in ovary wall, top of style and stigma (Fig. 2A: F2 to F5).

As for the full-length promoter, T1 seedlings of 5′-truncated GbSLSP-transgenic lines were GUS stained. As showed in Fig. 3, the seedlings of *F2* and *F3* had stronger GUS staining than those of *F4* and *F5* (Fig. 3A). The GUS staining was guard cell-specific in both young leaves and cotyledons of the seedling from *F2* and *F3* transformants, whereas for *F4*, this specificity was clearly visible only in the cotyledons (Figs. 3B & 3C). In the T1 seedlings of *F5* transformants, no guard cell-specific but a very week blade cell-diffused GUS staining was observed in both young leaves and cotyledons (Figs. 3B & 3C). The GUS staining strength in the T1 seedlings of all 5′-tructated GbSLSPs was similar to their parents, and weaker than that from the full-length *GbSLSP*.

GFP expression pattern in the young leaves of T0 transformants of all 5′-truncated *GbSLSPs* was similar to that of GUS staining except for *F4*, in which the GFP signal was clearly guard cell-specific whereas the guard cell-specificity of GUS staining was not very net (Fig. 2B: F2 to F5).

### 5′-UTR Plays an Important Role in the Determination of Tissue/organ-specificity and Strength of the Promoters in Transgenic Tobacco

Both full-length *GbSLSP* promoter and its 5′-truncated versions contained a 5′-UTR of 369 bp (from TSS to just upstream of the translation start codon “ATG”, Figs. 1 & 4A). In order to investigate the possible involvement of the 5′-UTR in the determination of tissue/organ-specificity and strength of promoter, we conducted PCR 3′-deletion of the full-length *GbSLSP* and one of its 5′-tructated versions, *F2*, the strongest guard cell-specific promoter, leaving only 9 bp of 5′-UTR just downstream of the TSS and generated promoters *F1-sh* and *F2-sh*, respectively (Fig. 4A). As for functional analysis of the full-length GbSLSP, we constructed F1-sh::GUS/GFP and F2-sh::GUS/GFP vectors and generated transgenic tobacco plants.

GUS staining of young leaves of T0 transformants showed that deletion of 5′-UTR from the full-length promoter GbSLSP not only decreased significantly the GUS activity, but also almost completely abolished its guard cell-preference in leaves (Fig. 2A: F1-sh ). In comparing with intact full-length GbSLSP, theF1-sh had weak and diffused GUS blue in the veins and blade cells (Fig. 2A: F1-sh vs. F1). Differently, deletion of 5′-UTR from *F2* did not clearly altered GUS expression pattern, retaining the guard cell-specificity, although the GUS activity was greatly reduced in the leaves (Fig. 2A:F2-sh). In the flowers of T0 transformant, *F2-sh* had only some anther slightly GUS-stained (Fig. 2A: F2-sh), whereas for *F1-sh*, strong GUS staining were present in the sepal, ovary wall and style top (Fig. 2A: F1-sh).

The GUS staining pattern of the T1 seedlings of 3′-deleted GbSLSPs transformants was overall similar to their parent lines, and the guard cell-specific staining was only seen in some of the seedlings from *F2-sh*, but not in those from *F1-sh* (Fig. 3A), both in young leaves (Fig. 3B) and cotyledons (Fig. 3C).

The GFP florescence detection of the young leaves of T0 transgenic tobacco showed that no clear GFP signal was detected in *F1-sh* transformants, but few guard cells had very weak GFP signal in *F2-sh* transgenic lines (Fig. 2B:F1-sh & F2-sh).

### The Guard Cell-specificity of *GbSLSPF2*, a 5′-trucated Version of *GbSLSP* Promoter, was Confirmed in Transgenic Arabidopsis

To study whether or not that the expression patterns of *GbSFLP* and its 5′-deleted versions seen in transgenic tobacco are reproducible in different plant taxa, we generated transgenic Arabidopsis T1 and T2 plants with 3 representative constructs, GbSLSP(F1)::GUS, GbSLSPF2 (F2)::GUS and GbSLSPF5 (F5)::GUS.

**Figure 5:**
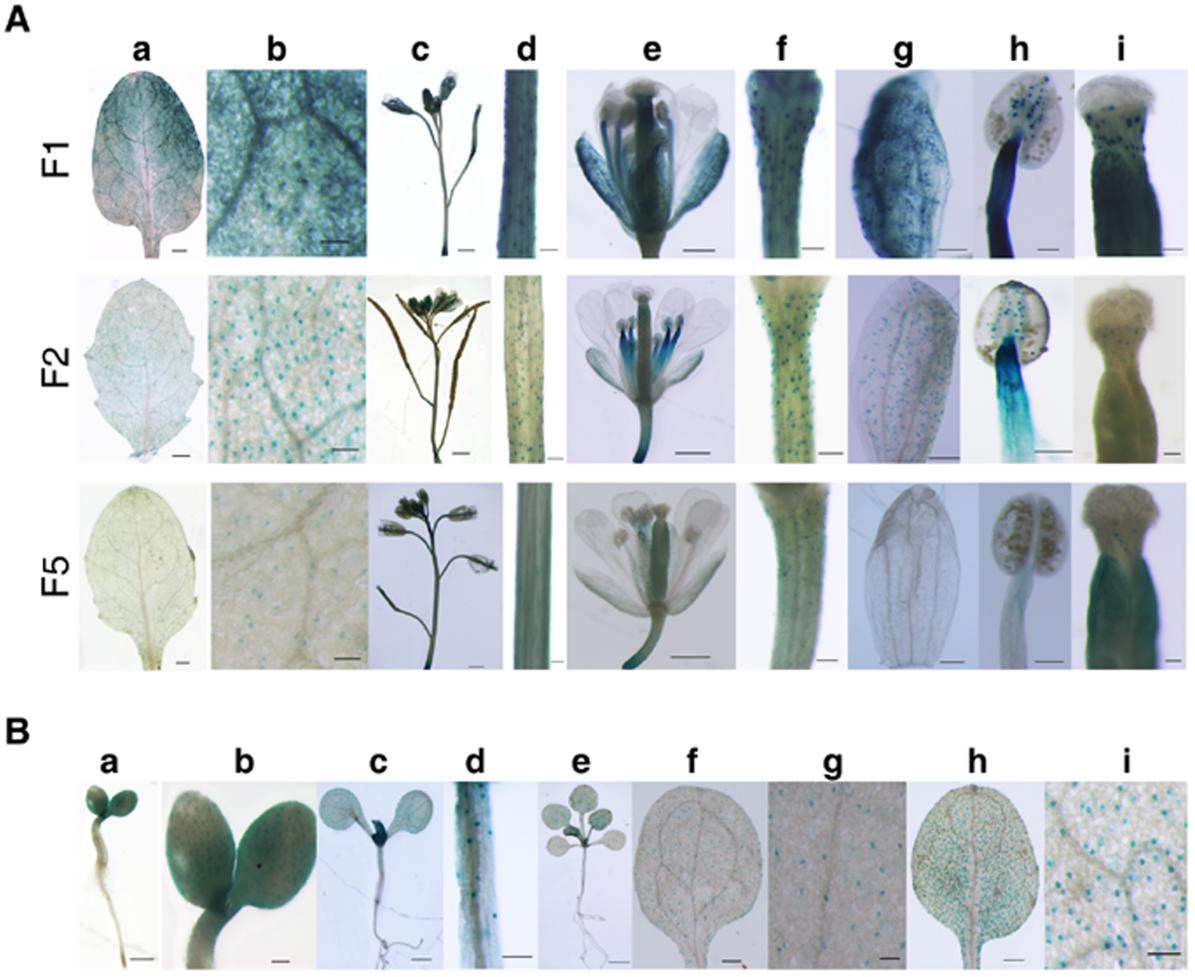
Histochemical GUS staining of transgenic Arabidopsis plants. A, GUS staining in different organs of the T1 transgenic plants. a, Rosette leaves, scale bars = 0.5 mm. b, Magnified view of a, scale bars = 0.1 mm. c, Inflorescence, scale bar = 1.0 mm. d, Inflorescence shoots, scale bars = 0.1 mm. e, Flowers, scale bars = 0.5 mm. f, Flower pedicles, scale bars = 0.1 mm. g, Sepals, scale bars = 0.1 mm. h, Stamen, scale bars = 0.1 mm. i, Styles, scale bars = 0.1 mm. B, GUS staining in different developmental stages of T2 seedlings of the plants transformed by GbSLSPF2. a, 3-day old seedling, scale bar = 0.5 mm. b, Magnified view of a, scale bar = 0.2 mm. c, 7-day old seedling, scale bar = 1.0 mm. d, Hypocotyl of c, scale bar = 0.2 mm. e, 15-day old seedling, scale bar = 1.5 mm. f, Cotyledon of e, scale bar = 0.3 mm. g, Magnified view of f, scale bar = 0.1 mm. h, Rosette leaf of e, scale bar = 0.3 mm. i, Magnified view of h, scale bar = 0.1 mm.

As what seen in transgenic tobacco, the full-length GbSLSP drove GUS gene to express strongly in the developing and fully expanded rosette leaves (young leaves), inflorescence shoots, flower pedicles, sepals, stamen, and styles in Arabidopsis T1 transformants, and the expression was more pronounced in guard cells than other epidermic cells (Fig. 5A: F1). The *F2::GUS* also expressed in above organs, but exclusively in their guard cells (Fig. 5A: F2). In *F5::GUS*-transformants, GUS blue was almost absent in the sepals, stamen, but present moderately in inflorescence shoot, flower pedicles and styles, weakly in young leaves (Fig. 5A: F5). The GUS expression pattern of *F5* in Arabidopsis T1 transformants was similar to that in transgenic tobacco, but the expression was stronger, and in particular in the inflorescence shoot, style and rosette leaves.

GUS staining of Arabidopsis T2 seedlings of *F2* transformants showed that the guard cell-specificity conferred by *F2* was retained, and the expression seemly regulated by developmental stages (Fig. 5B). In 3-d seedlings, a strong GUS blue appeared in cotyledons and up-part of hypocotyl adjacent to cotyledon with a guard cell-preferred manner (Fig. 5B: a & b). However, In 7-d (Fig. 5B: c & d) and older seedlings (Fig. 5B: e to i), the GUS expression became guard cell-specific in the hypocotyl, cotyledon and young leaf.

## Discussion

### Mining for Gene and its Major Regulatory Element(s) of a Specific Interest

Rapid increasing DNA and mRNA sequence databases provide very rich resources for mining genes and their regulatory element(s) of a specific interest [59], [61]. In order to isolate guard cell-specific promoter for further use in stomata study and in the improvement of crops adaptation to environments, we scanned available DNA and mRNA databases with two criteria: A, presence of the guard cell-specific *cis*-acting element “(T/A)AAAG” [35] approximate to the transcription start site (TSS) in the regulatory region of a gene. B, the protein deduced coded by the gene is involved in stomatal density, distribution, development and/or movement. This scanning led to target an up-land cotton gene “*GhSCFP*” which was cloned and named by Hou et al. [54], [55]. We separately online analyzed in detail the regulatory region and deduced protein of *GhSCFP* by using SoftBerry (http://linux1.softberry.com), PLACE [60] and Blast (NCBI), respectively. The analysis of the regulatory region disclosed that there existed more than one “(T/A)AAAG” elements near TSS (date not shown), which meets our first selection criteria. Blasting of the deduced protein revealed its sharing more than 85% homology with subtilisin-like serine proteases (subtilases) from Arabidopsis, potato and rice (date not shown). Thus, the “*SCFP”* was renamed “*SLSP*”. In Arabidopsis, *SDD1*, one of 56 copies of subtilases [62], was contributed to stomatal development, density and distribution [50], and thus satisfies our second selection criteria. Therefore, we cloned the regulatory region upstream of the translation start point of *SLSP* from sea island cotton (*Gossypium barbadense*) [52]. As predicated, our cloned promoter region of *GbSLSP* had the TATA-Box, TSS and their around sequences almost identical to those of GhSCFP (date not shown), and contained 10 copies of Dof1 elements (grey-boxed in Fig. 1), including 3 copies of guard cell-specific *cis*-elements, TAAAG, approximate to TSS, 1 in sense strand (−229) and 2 in antisense one (−110, −479) (grey-boxed and underlined in Fig. 1). The full-length *GbSLSP* indeed directed strong guard cell-preferred expression of GUS and GFP reporter genes in both transgenic tobacco (Fig. 2: F1;Fig. 3: F1) and Arabidopsis (Fig. 5: F1). These results suggest that it would be easy to mine available DNA and mRNA sequence databases for genes and their major regulatory element(s) of a specific interest if the “probe” and probing criteria are appropriate. The results suggest also that the *cis*-element (T/A)AAAG approximate to TSS identified by Plesch and colleagues [35] and used in this experiment is an appropriate probe for guard cell-preferred and/or -specific promoter mining.

### Relationship between *cis*-acting Element (T/A)AAAG and Guard Cell-specific Expression

*Cis*-acting element (T/A)AAAG of promoters is well-known as the target site of Dof1 zinc finger transcription factors [63] and the TAAAG in potato *Kst1* promoter was found to play a critical role in guard cell specific gene expression [35]. However, a grand body of promoters contain (T/A)AAAG elements, usually in more than one copy, but they are not guard cell-specific, even not guard cell-preferred [44], such as, *Bnfs*, *Bofs*, *Bpfs*, *Bcfs* [59], *ATA7* [61] and even the full length of potato AGPase promoter [32] from which the guard cell-specific element TAAAG was identified [35]. Thus the relationship between (T/A)AAAG elements and guard cell specific gene expression is beyond the simplicity.

Müller-Rober et al [32] reported that the full length of potato AGPase promoter which contained 10 (T/A)AAAG elements didn’t drive guard cell-specific expression of the GUS reporter gene, but its 300 bp 5′-truncated version which retained only 5 elements could specifically expressed in the guard cells. They postulated that the (T/A) AAAG elements far away from the TSS might not work for the guard cell specificity. This position effect was also observed in *CYP86A2* promoter in which the presence of 2 more (T/A) AAAG elements at −805/−883 abolished the guard cell specificity [26]. Our results showed that the full-length promoter *GbSLSP* (F1) contained 3 more (T/A)AAAG elements (including 1 guard cell-specific one at −479) at 5′ distal than *F2*, (Figs. 1 & 4A) and directed only guard cell-preferred expression of GUS and GFP reporter gene in both transgenic tobacco (Fig. 2: F1; Fig. 3: F1) and Arabidopsis (Fig. 5A: F1), whereas the *F2*, 5′-truncated version of *GbSLSP,* did confer the guard cell-specific expression of the reporter genes (Fig. 2: F2; Figs. 3 & 5: F2). This suggests that the (T/A)AAAG elements, especially TAAAG, proximal to TSS might determine guard cell specific expression of the gene, whereas those far away from the TSS might not only do not work for, but also impede the guard cell specificity. Neininger et al [64] and Dorbe et al [65] observed that in spinach and tobacco NIR gene promoters, the sequences close to their TSS were sufficient to confer nitrate-responsive increases in reporter enzyme activity. Besides the position effect of (T/A)AAAG relative to the TSS, the distance in (T/A)AAAG clusters and/or the distance between clusters and coding region may affect guard cell specific expression, for which the *Kat1* [33] is exampled. Galbiati and co-workers [26] suggested that a cluster of at least 3 copies of (T/A)AAAG elements located on the same strand within a region of 100 bp of *AtMYB60* be decisive to guard cell-specific expression of the promoter. In our experiment, the *F4* which contains a cluster of 3 (T/A)AAAG elements located on the different strands (2 in sense strand and 1 in antisense one) in a region of ca. 100 bp relative to TSS (Fig. 1) was “true” guard cell-specific (Fig. 2: F4; Fig. 3: F4), and removal of one distal TAAAG element from the cluster (Figs. 1 & 4A) resulted in complete abolishment of the guard cell specificity (Fig. 2:F5; Figs. 3 & 5:F5). Thus, if the cluster were decisive to guard cell-specific expression of the promoter, the distal copy of element in the cluster would play an essential role without the necessity of same strand location of the (T/A) AAAG elements in the cluster.

In addition to their position and/or distance effects, the (T/A)AAAG element copy number may have some effects on the guard cell-specific expression. Yang and colleagues [44] observed that *AtMYB61* which contains 29 (T/A)AAAG elements had lower expression in guard cell than *AtACT7* which has only 23 (T/A) AAAG elements, and block mutagenesis of the central TAAAG motif on the sense strand in the 8 TAAAG motifs-containing region (−861 bp to −224 bp) of GC1 promoter did not affect reporter expression in guard cells. Thus they thought that it was not the number or mutation of several (T/A) AAAG elements that could affect the expressive activity in guard cells. However, in our experiment, progressively reducing the number of (T/A) AAAG elements proximal to the TSS, i.e. from F2 (containing 7 copies of (T/A) AAAG elements) to F5 (containing only 2 copy), greatly decreased the expressive activity of both GUS and GFP reporter genes in the guard cells of transgenic tobacco (Fig. 2:F2 to F5) and Arabidopsis (Fig. 5A). Thus, the Dof elements in the strict guard cell-specific promoters seemly have an additive effect on the gene expression strength in guard cells, which was also observed by Cominelli and colleagues in a “true guard cell-specific promoter”, *AtMYB60* [42]. This different effects of (T/A) AAAG copy number may be contributed to much larger distance of the Dof elements relative to TSS in *GC1* promoter (−861 bp to −224 bp) than in our *F2* to *F5* (−262 bp to −44 bp) and than in *AtMYB60* minimal promoter region (−196 bp), because the Dof elements far away from the TSS may enhance the guard cell expression activity, but decreased the guard cell specificity as discussed above. Of course, the (T/A)AAAG element alone may not completely explain why guard cell-specific promoters exhibited guard cell-specific expression, as discussed by Yang and colleagues [44], demonstrated by Cominelli et al [42] and revealed by our 3′-deletion of the *GbSLSP* which will be discussed in the following.

### Roles Played by 5′-UTR in the Determination of the Guard Cell Expression Activity and Specificity

It is well known that the 5′-untranslated region (5′-UTR) takes an important part in regulating gene expression at transcriptional and post-transcriptional levels [66], [67]. This regulation was mostly reported concentrated on gene expression strength, i.e. increasing or decreasing downstream gene’s expression. For example, the 5′-UTRs of *ntp303* [68], *OsADH* [69] and *OsGluc* [70] enhanced markedly endogenous gene and/or GUS reporter gene expression, whereas the 5′-UTR of *LAT59* greatly decreased mRNA yields [71]. In our experiment, the 5′-UTR of *GbSLSP* promoter affected not only the gene expression strength, but also the gene expression specificity. Removal of 359 bp out 369 bp 5′-UTR from full-length *GbSLSP* by 3′-deletion (Fig. 4A), significantly decreased the expression strength of both GUS and GFP reporter genes in transgenic tobacco (in Fig. 2: F1-sh vs. F1; Fig. 3: F1-sh vs. F1; Fig. 4B), and the same 3′-deletion in the strong guard cell-specific promoter *F2* (Fig. 4A), not only reduced the expression activity (Fig. 4B), but also completely abolished the guard cell-specificity of reporter genes (Fig. 2: F2-sh vs. F2; Fig. 3: F2-sh vs. F2). From these comparisons (F1 vs. F1-sh, F2 vs. F2-sh) and comparisons in the previous section (F1 vs. F2 to F5), we can see that the 5′-UTR in the *GbSLSP(s)* acts as an enhancer in one hand, and takes part in guard cell-specific expression of the reporter genes in the other hand.

In summary, we isolated a 993-bp promoter region upstream of the translation start point of subtilisin-like serine protease (subtilase) gene from sea island cotton, and demonstrated that 5′-end truncated versions of the promoter, *F2 to F4*, could drive GUS and GFP reporter genes to express exclusively and strongly in the guard cells of both transgenic tobacco and Arabidopsis plants, while the full-length *GbSLSP* directed high level guard cell-preferred expression. We revealed that the guard cell specificity and expression strength of the promoters were coordinately controlled by 5′-untranslated region (5′-UTR) and a cluster of at least 3 copies of (T/A)AAAG elements within a region of about 100 bp relative to transcription start site (TSS). We are aware that in order to better use these new “true” guard cell-specific promoters to manipulate gene expression in guard cells for physiological and biochemical studies and for biotechnological improvement of crop plants, further work is needed to investigate whether the guard cell specificity and strength of these new promoters are regulatable, and if yes, what is the major regulator(s).
